# Gonadoblastoma with Dysgerminoma in a Phenotypically Turner-Like Girl with 45,X/46,XY Karyotype

**DOI:** 10.4274/jcrpe.2022

**Published:** 2015-12-03

**Authors:** Özge Yüce, Esra Döğer, Nurullah Çelik, Hamdi Cihan Emeksiz, Mahmut Orhun Çamurdan, Aysun Bideci, Peyami Cinaz

**Affiliations:** 1 Gazi University Faculty of Medicine, Department of Pediatric Endocrinology, Ankara, Turkey

**Keywords:** 45, X/46, XY gonadal dysgenesis, gonadoblastoma, dysgerminoma

## Abstract

Individuals with 45,X/46,XY karyotype are at increased risk for germ cell tumor development. We report a case with a diagnosis of 45,X/46,XY gonadal dysgenesis who presented with short stature, physical stigmata of Turner syndrome. Her pubertal development was at Tanner stage 3. At follow-up, bilateral prophylactic gonadectomy was performed when considering the risk factors. Pathological assessment was consistent with gonadoblastoma in the left gonad, and dysgerminoma and gonadoblastoma in neighboring areas in the right gonad. The karyotype analysis of the right and left gonadal tissues reveled 45,X[97,3]/46,XY[2,7] and 45,X[92,7]/46,XY[4,5]/47,XYY [2,8] mosaic, respectively. The clinical management of such patient should be individualized according to the present risk factors. Additionally, signs of estrogenization like advanced breast development always suggest the possible presence of germ cell tumor.

**WHAT IS ALREADY KNOWN ON THIS TOPIC?**

Patients with gonadal dysgenesis, particularly those carrying Y chromosomal material in the gonadal tissue, are at an increased risk for germ cell tumor development.

**WHAT THIS STUDY ADDS?**

In this context, AZFc gene deletion on peripheral blood lymphocytes DNA detected in our patient (Y-chromosomal microdeletion of the AZFc region) may contribute to germ cell tumour development as a risk factor. This can be expected when considering the role of AZF genes in gonadal cell differentiation by providing Y-chromosome stability during early embryogenic development.

## INTRODUCTION

Gonadal dysgenesis (GD) is characterized by the abnormal development of the foetal gonads due to structural or numerical anomalies of the sex chromosomes or mutations in genes involved in the formation of the urogenital ridge and in sex determination of the bipotential gonad (1). Patients with GD, particularly those carrying Y chromosomal material in the gonadal tissue, are at an increased risk for germ cell tumour (GCT) development (1,2). One particular type of GD, 45,X/46,XY, has an estimated GCT development risk of 15% (1). Patients with a mosaic 45,X/46,XY karyotype have a wide range of phenotypic manifestations, ranging from females with Turner stigmata to males with mixed GD and/or ambiguous genitalia to almost a normal male phenotype (3). Tumour risk was found to be significantly associated with clinical phenotype and revealed to be very high in patients with an ambiguous phenotype (4). However, in 45,X/46,XY Turner-like patients without signs of virilisation, the tumour risk is low (5).

Here, we report a phenotypically Turner-like patient who had a 45,X/46,XY karyotype with a Yq microdeletion. The patient developed two different types of GCTs at each gonad.

## CASE REPORT

A 13-year-old female patient presented to our endocrinology clinic for evaluation of short stature. She had been shorter than her peers throughout her life. She was the third child of non-consanguineous parents and her two siblings were normal.

Adjunct to short stature, her examination revealed physical stigma for Turner syndrome (TS) such as a short/webbed neck, cubitus valgus, and short 4th metacarpal bones. She stood 139.3 cm [-2.57 standard deviation (SD)] and weighed 41.2 kg (+2 SD). Both breast and pubic hair development were consistent with Tanner stage 3. Her external genitalia appeared completely female. Bone age determined by Greulich and Pyle atlas was 13 years.

She had a follicle-stimulating hormone level of 121 IU/L, luteinizing hormone (LH) level of 28.8 IU/L, and an estradiol level of 28.6 pg/mL. Complete blood count, celiac markers, thyroid function tests, insulin-like growth factor-1, and insulin-like growth factor-binding protein-3 were all within normal range. She had a uterus of 9x14x33 mm in size, but her ovaries could not be visualized by ultrasonography. Further magnetic resonance imaging to evaluate internal genital organs revealed a right gonad with a size of 22x14x19 mm, an invisible left gonad, and a hypoplastic uterus. The chromosomal study of peripheral blood lymphocytes (PBL) with GTG-banding analysis of 55 metaphases showed a 45,X[30]/46,XY[25] karyotype. Y-derived sequences (SRY, AZF, Ycen, and Yqh) were analysed by polymerase chain reaction (PCR); they were all present except for microdeletions in AZF regions (SY254, SY255) on PBL DNA.

Due to an increased malignancy risk to the gonads, an explorative laparotomy and bilateral gonadectomy were performed. During explorative laparotomy, she was found to have a streak gonad on the left side and a 3-cm irregular nodule in the ovarian fossa on the right. The capsules of the gonadal tissues were intact, and the gonads were successfully removed. The frozen sample of the right nodule was consistent with dysgerminoma. On inspection, the uterus and fallopian tubes were normal, and there was no evidence of malignancy or abnormality in the pelvic organs. Histopathological examination of the left streak gonad revealed a gonadoblastoma. In the right gonad, together with a normal ovarian stroma, centrally located dysgerminoma and gonadoblastoma in neighbouring areas were identified. Twenty-three metaphases were analysed in each gonad. The karyotype of the right and left gonadal tissues were 45,X[97,3]/46,XY[2,7] and a 45,X[92,7]/46,XY[4,5]/47,XYY[2,8] mosaic, respectively. The microdeletion in the AZF region was detected by using PCR analysis.

The patient was discharged one week later, after an uneventful postoperative course. She then consulted with paediatric oncology. Based on their recommendations, computed tomography scans of the chest, abdomen and pelvis were performed for metastatic evaluation and did not reveal an abnormality. GCT markers (lactate dehydrogenase, human chorionic gonadotropin, alpha-fetoprotein) were measured, and all were within normal limits. After the first oncological evaluation, a follow-up plan was designed for every 3 months during the first year and every 6 months thereafter. At the same time, hormone replacement therapy was started to maintain secondary sexual characteristics and to prevent bone loss.

The patient and especially her parents had numerous questions regarding fertility and gender identity, and therefore, she was referred to an adolescent psychiatrist and a gynaecologist. Her gender identity was that of a woman, and she was worried about having children. Future fertility treatment options, such as adoption and oocyte-donor in-vitro fertilization (IVF), were also discussed.

Her regular follow-up has been ongoing in outpatient clinics of related departments nearly for the past postoperative 2 years and no complications have been noted.

## DISCUSSION

The mosaicism of 45,X/46,XY is an important component of sex chromosome disorders of sexual development (DSD) and occurs with an incidence of 1.5 per 10,000 (6). X/XY individuals have a wide range of cytogenetic, clinical, and histopathological features. They present as a Turner-like syndrome having bilateral streak gonads with Müllerian structures and TS stigmata; as mixed GD having unilateral testis and a contralateral streak gonad and Müllerian structures; as a male having bilateral testes (any combination of dysgenetic or normal testes) but with Müllerian structures and signs of decreased virilisation; or as a normal male having bilateral testes and normal male genitalia (7,8,9). Its clinical importance is due to the increased risk of GCT development, that is, seminomas/gonadoblastoma/dysgerminoma and non-seminomas (e.g. embryonal carcinoma, yolk sac tumour, choriocarcinoma, and teratoma) (2).

GCTs most often occur in a subgroup of DSD patients. The risk among subgroups of DSD is heterogeneity, and DSD can also be classified into different levels as high, intermediate, low, and unknown. GCT is associated with gonadal dysgenesis, hypervirilisation in 46,XX individuals, and undervirilisation (incomplete masculinisation, ambiguous or female phenotype) in 46,XY individuals (2,10).

The presence of the Y chromosome or molecular evidence of a Y-derived sequence serves as the risk factor for the origin of GCTs in those who have a dysgenetic gonad. This has been particularly related to the presence of a specific part of the Y chromosome that is known as the gonadoblastoma region of the Y chromosome (GBY) and candidate genes that are located in this chromosomal fragment (11). Of these, the testis-specific protein on Y (TSPY) is one of the most interesting. Its aberrant expression has been related to increased proliferation of germ cells and oncogenic activity (12). Furthermore, sex-determining region Y (SRY) gene abnormalities (e.g. mutations, inadequate function) are associated with an increased risk of GCT development due to poorly differentiated gonadal tissue (2,13). Bianco et al (14) reported that the presence of the SRY gene increases the risk of gonadoblastoma development in mosaic TS patients by predisposing modifications in the gonadal microenvironment.

Besides the aforementioned genetic risk factors, AZFc gene deletion on PBL DNA detected in our patient (Y-chromosomal microdeletion of the AZFc region) may contribute to GCT development as a risk factor. This can be expected when considering the role of AZF genes in gonadal cell differentiation by providing Y-chromosome stability during early embryogenic development (15,16,17). Additionally, it is also reported that microdeletions in this specific gene loci increase the risk of GCT development particularly in infertile males (18).

It ıs well known that patients with 45,X/46,XY GD have a high incidence of Y-chromosome microdeletions and that there is a possible association with the severity of the phenotype (17). However, the genetic contribution of these microdeletions to the risk of developing malignancy has not been reported in the literature.

In 45,X/46 XY Turner girls without signs of virilisation, tumour risk is low and tumour development is generally expected in postpubertal ages (5). Gonadoblastoma together with dysgerminoma has been presented as case reports in 45,X/46,XY DSDs (19,20,21). Our case is an example of this and was diagnosed in a patient aged 13 years. This was a surprise to us because the time of emergence of malignancy was early when considering risk factors such as age, phenotypic features, and the presence of a low-level XY cell line in her gonadal tissue sample. We suggest that this could be related to the detected AZF gene deletion along with known risk factors.

Due to the increased risk of GCT development and the high prevalence of this tumour in mosaic patients, prophylactic gonadectomy has recently been recommended. However, new guidelines recommend a more conservative approach because of the decreased risk of GCT development in a histologically more mature gonad. Therefore, maturation stage and functionality of the gonad are of fundamental importance in the decision for gonadectomy (2,4). In female patients with the 45,X/46,XY karyotype, the removal of gonads are highly recommended because germ cells of a streak gonad are likely to undergo tumoral transformation, and even with a conservative approach, fertility will not be an issue for such cases. Gonadectomy can be postponed in patients who are reluctant or who are at an increased risk for surgery (2,4,5). However, gene abnormalities (deletion, duplication, etc.) and their role in gonadal differentiation need to be considered when deciding on the time of surgery.

Consequently, there are several potential genetic risk factors for GCT development in cases with mosaic karyotype. The risk for malignant transformation may also occur early in life and may increase with age. Therefore, in the event of detection of any genetic disorder involved in cell differentiation and/or of a clinical suspicion for GCT development, gonadectomy should not be postponed in girls with a mosaic karyotype despite the low risk of GCT development in such cases.
